# Production of extracellular vesicles with light‐induced proton pump activity by proteorhodopsin‐containing marine bacteria

**DOI:** 10.1002/mbo3.808

**Published:** 2019-02-22

**Authors:** Yong Min Kwon, Ajit Kumar Patra, Hiroshi Xavier Chiura, Sang‐Jin Kim

**Affiliations:** ^1^ National Marine Biodiversity Institute of Korea Seocheon Korea; ^2^ Genetics Research Group Centre for Earth Surface System Dynamics, Atmosphere and Ocean Research Institute, the University of Tokyo Kashiwa, Chiba Japan; ^3^ Department of Bioregulation and Biointeraction, Laboratory of Molecular and Cellular Biology Graduate School of Agriculture, Tokyo University of Agriculture and Technology Tokyo Japan; ^4^ Marine Biotechnology Research Center Korea Institute of Ocean Science & Technology Pusan Korea; ^5^ BJC Co. Ltd. Incheon Korea

**Keywords:** extracellular vesicles, flavobacteria, light‐induced proton pump activity, metagenomics, proteorhodopsin, S13EVs

## Abstract

The production and release of extracellular vesicles (EVs) is a common process occurring in various types of bacteria. However, little is known regarding the functions of EVs derived from marine bacteria. We observed that during cell growth, *Sediminicola* sp. YIK13, a proteorhodopsin (PR)‐containing marine flavobacterium, produces EVs (S13EVs). Transmission electron microscopy showed that *Sediminicola* sp. YIK13 released two spherical vesicle types, with mono‐ and/or bi‐layered membranes, in the culture. Interestingly, the S13EVs have an orange pigment, indicating the presence of putative carotenoid and PR pigments ascribed to the parental cells. The S13EVs demonstrated the same PR‐derived absorption peak spectrum and light‐induced proton pump activity as the parental cells. Western blot (immunoblot) analysis of the S13EVs revealed the presence of PR. We confirmed the 16S rRNA gene, *pro* gene, and genes required for chromophore retinal synthesis, namely *blh* and *crtI*, in the DNA packaged into these vesicles. In addition, by metagenomic sequencing, we found microbial rhodopsin‐related genes in vesicles derived from natural aquatic environments. Our results suggest that EVs as well potentially pursue horizontal gene transfer of diverse microbial rhodopsin genes in marine ecosystems.

## 1 INTRODUCTION

Extracellular vesicle (EV) production is a ubiquitous process in all domains of life, namely Eukarya, Bacteria, and Archaea (Deatherage & Cookson, 2012). Gram‐negative bacteria release cell‐associated constituents, including proteins, lipoproteins, lipopolysaccharides, and genetic materials, through EVs, which are also known as outer membrane vesicles (OMVs) (Kulp & Kuehn, 2010; Manning & Kuehn, 2013). In gram‐negative bacteria, the budding and release of the outer membranes produce EVs (small spherical structures that are 10–300 nm in diameter); however, the mechanisms of EV production are still unclear (Kulp & Kuehn, 2010). Several previous studies have proposed that these biological organelles are involved in toxin delivery (Chutkan & Kuehn, 2011; Kesty, Mason, Reedy, Miller, & Kuehn, 2004; Wai et al., 2003), biofilm formation (Schooling, Hubley, & Beveridge, 2009; Yonezawa et al., 2009), quorum sensing (Mashburn & Whiteley, 2005), defense against antimicrobials (Manning & Kuehn, 2011), nutrient acquisition (Gorby et al., 2008), horizontal gene transfer (Renelli, Matias, Lo, & Beveridge, 2004; Yaron, Kolling, Simon, & Matthews, 2000), and ATP transfer (Pérez‐Cruz, Delgado, López‐Iglesias, & Mercade, 2015). To date, most studies on pathogenic and nonpathogenic gram‐negative bacteria have shown evidence of EV production under various culture conditions and even in varying natural environments. Recently, Biller et al. (2014) first showed that several strains originating from the dominant genus *Prochlorococcus* of the marine cyanobacteria produce large amounts of membrane vesicles (MVs) containing proteins, DNA, and RNA, suggesting that marine phototrophic bacteria produce MVs both in situ and in vitro. Moreover, Biller et al. (2014) proposed functional roles of marine MVs, including cellular communication, horizontal gene transfer, carbon cycling, and phage defense. However, very little is known about the functions and presence of EVs produced by marine bacteria.

In this study, we isolated a marine photoheterotrophic flavobacterium, *Sediminicola*, from a marine environment to test its EV production. Flavobacteria, the major clade of the phylum Bacteroidetes (previously known as the Cytophaga‐Flavobacterium‐Bacteroides group), occupy an important position in the marine photometabolic system (Venter et al., 2004). *Sediminicola* sp. YIK13 is one of the several strains containing proteorhodopsin (PR) isolated from marine sediments (Kwon, Kim, Jung, & Kim, 2016). PR phototrophy is a likely source of significant microbial processes in marine environments, where light‐driven proton pumps convert sunlight to proton gradients that generate energy for processes, such as cell growth and maintenance (Béjà et al., 2000; Martinez, Bradley, Waldbauer, Summons, & DeLong, 2007). Since the discovery of PR in 2000, research has revealed the abundance of PRs, their diversity, and their biochemical functions in the global ocean (Béjà et al., 2000; Béjà, Spudich, Spudich, Leclerc, & DeLong, 2001; Martinez et al., 2007; Sabehi et al., 2003, 2005; de la Torre et al., 2003).

Here, we reported the production of EVs derived from a marine flavobacterium, *Sediminicola* sp. YIK13, and attempt to characterize their physiological features. We directly observed the PR‐mediated pump activity, absorption spectrum, and immunoblot of the orange‐pigmented EVs from *Sediminicola* sp. YIK13 (S13EVs), which revealed the presence of carotenoid and PR proteins like those in the parental cells. The DNA packaged into the S13EVs permitted investigations into the PR and retinal synthesis genes. Furthermore, we investigated the presence of diverse microbial rhodopsin genes in EVs derived from natural environments, based on shotgun sequencing‐generated metagenomes. To the best of our knowledge, no previous study has identified these genes in EVs. Thus, it is important to understand the role that EVs containing PR genes play in natural environments, as well as the functions of the PR gene and protein within the EVs.

## 2 MATERIALS AND METHODS

### 2.1 Strains and cultivation

*Sediminicola* sp. YIK13 and *N. antaticus* AKS622^T^ strains were isolated from tidal flat sediments on Yeongheung Island at the coast of the West Sea, Republic of Korea (Kwon et al., 2016) and from a glacial ice core at the coast near King Sejong station on King George Island, Antarctica (Kwon, Yang, Kwon, & Kim, 2014), respectively. The YIK13 and AKS622^T^ strains were routinely cultured on Marine agar 2216 (MA; Difco, USA) or ZoBell medium (ZB; 5‐g peptone, 1g yeast extract, 0.01g FePO_4_ per liter of 20% distilled water, and 80% aged seawater) and incubated at 30°C and 15°C, respectively, with continuous shaking at 120 rpm.

### 2.2 EV purification and production ratio measurements

EVs were purified from the 5 L of YIK13 and AKS622^T^ strain culture medium according to methods previously described (Choi et al., 2015). Briefly, YIK13 cells grown to the late stationary phase were harvested by centrifugation at 7,500 × *g* for 40 min at 4°C. Supernatants were sequentially filtered through 0.45‐μm and 0.2‐μm membrane filters (Advantec, Japan), and concentrated with a Millipore Ultra‐filtration system using a 100 kDa cutoff membrane (Millipore, USA). The concentrated samples were centrifuged to collect the vesicles as a pellet by ultracentrifugation (Hitachi, Japan) at 88,000 × *g* for 40 min at 4°C and were resuspended overnight at 4°C in 500 μl of 1 × sterilized TBT buffer (100 mmol/L Tris‐HCl, 100 mmol/L NaCl, and 10 mmol/L MgCl_2_; pH 7.4) at slow rotation. Vesicle samples were treated with 30 μg/ml of both DNase and RNase A (Sigma, USA) for 30 min at 37°C, and nucleases were heat inactivated at 70°C for 10 min. Vesicle samples were then purified by ultracentrifugation in 35% CsCl density gradients, and their buoyant densities were calculated at 25°C, followed by dialysis against 1 × TBT buffer to remove CsCl from the recovered EVs. To verify that purified EV samples were cell‐free, 10‐μl aliquots of the samples were plated in duplicates onto MA plates and incubated at 30°C for 7 days. The absence of any viable or non‐viable bacteria was further verified by electron microscopy. Purified EV samples were preserved at 4°C for further experimentation.

Samples to determine the vesicle‐to‐cell ratio were collected from 50‐ml cultures at 0, 6, 12, 24, 36, 48, 60, and 72 hr. Briefly, 2 ml of each culture was fixed with 2.5% (v/v) glutaraldehyde for cell counting. Remaining cell cultures were centrifuged, filtered through a 0.2 μm syringe filter, and then vesicle samples were resuspended after ultracentrifugation under the same conditions as described above. The fixed cells and EV numbers were analyzed using a qNano (Izon Science, New Zealand) instrument according to a previously described method (Choi et al., 2015; Kwon et al., 2016). Cells and EVs were diluted 1,000‐fold into 0.2‐μm filtered 1 × TBT buffer and were measured using NP1000 (500–2000 nm) and NP150 (75–300 nm) membranes, respectively. Each measurement was recorded at ≥500 pulses and repeated three times. Calibration was performed using CP1000 for cells and CP100 for vesicles under the same conditions as the standard control. Data were analyzed using the Izon control software (version 2.2).

### 2.3 EV size and concentration

The size and concentration of purified EVs were assessed by two methods: qNano and NTA (Malvern, UK). The qNano analysis was performed as described above. NTA was performed on NanoSight NS300 equipped with a 405‐nm violet laser and a CMOS camera (Hamamatsu Photonics, Japan). Purified EVs were diluted 1,000 fold with 0.2‐μm filtered 1 × sterilized PBS buffer, and each sample was loaded into a flow‐cell top‐plate using a syringe pump. All videos of 30–60‐s duration at 25°C were recorded and processed using NTA software (version 3.1). All measurements were repeated three times.

### 2.4 Electron microscopy observation

Electron microscopy observation of two bacterial cells and purified EVs was performed according to a previously described method (Choi et al., 2015). The YIK13 grown for 6, 24, and 72 hr as well as AKS622^T^ grown for 72 hr were collected and pre‐fixed with 2% glutaraldehyde for 4 hr as well as post‐fixed with 1% osmium tetroxide for 2 hr. Samples were dehydrated with a series of graded ethanol solution, starting with 30%, 50%, 70%, 80%, and 90% along with three exchanges of anhydrous 100% and air dried after final dehydration in tetramethylsilane (Sigma, USA). Samples were sputter‐coated with gold and then examined by SEM (JSM‐840A, Japan) at an acceleration voltage of 5 kV and a magnification of 15,000×. EVs were placed on carbon‐coated Formvar grids (EMS, USA) and negatively stained for 10 s with 1% uranyl acetate. The grids were observed by TEM (JEM1010, Japan) at an acceleration voltage of 80 kV and a magnification of 50,000×.

### 2.5 Measurement of light‐driven pump activity and spectral analysis

Proton pump activity was measured according to a previously described method (Kwon et al., 2016). Cells grown for 72 hr and purified EVs were diluted to a concentration of 2 × 10^9^ ml^−1^ and 2 × 10^11^ ml^−1^, respectively using the qNano method described above. These samples were placed in dark conditions and then illuminated at an intensity of 100 W/m^2^ using a short‐wave cutoff filter (>440 nm, Sigma Koki SCF‐50S‐44Y, Japan) in combination with a focused convex lens and heat‐protected (1% CuSO_4_) filter. The pH values were monitored using a computerized Mettler Toledo S220 pH meter. Measurements were repeated under the same conditions after adding carbonylcyanide‐m‐chlorophenylhydrazone (CCCP) to obtain a final concentration of 10 μmol/ml.

Absorption spectra of two bacterial cells and S13EVs were measured using a UV‐vis spectrophotometer (Thermo Fisher Scientific GENESYS 10S, USA). Spectra were scanned in 0.5‐nm wavelength steps from 300 to 800 nm.

### 2.6 Immunoblot analysis

The cytoplasmic, inner membrane, and outer membrane were purified by a previously described method (Kesty et al., 2004). Whole cells and EVs were suspended in TN buffer (10 mmol/L Tris‐HCl and 50 mmol/L NaCl; pH 8.0) with a protease inhibitor cocktail (Sigma, USA) and then disrupted five times by ultrasonication (Branson Sonifier 550) at 20% amplitude for 5 s. Protein concentrations were quantified using the Bradford Protein Assay (Bio‐Rad, USA). Proteins (10 μg for Coomassie blue staining and 20 μg for immunoblot) from whole cell, cytoplasmic, inner membrane, outer membrane, and EVs were analyzed using 12% SDS‐PAGE and transferred onto a nitrocellulose membrane. The membrane was blocked in Tris‐buffered saline with Tween‐20 (TBST: 20 mmol/L Tris‐HCl, 137 mmol/L NaCl, and 0.2% Tween‐20; pH 7.6) containing 5% skimmed milk for 30 min and was then incubated for 2 hr with anti‐*Sediminicola* sp. PR_Arg71‐Tyr86_ peptide rabbit serum (1:500 dilution; AbFrontier, Korea). The membrane was washed twice in TBST and then incubated for 1 hr with AP‐conjugated secondary antibodies (1:1,000 dilution; GenDEPOT, USA). After washing with TBST, the membrane was developed using BCIP/NBT (Sigma, USA).

### 2.7 Epifluorescence microscopy and PCR amplification

S13EVs containing DNA were counted using a Zeiss epifluorescence microscope after staining the samples with SYBR gold (final SYBR gold concentration, 100×) according to a previously described method (Choi et al., 2015). S13EVs were pelleted from purified samples by ultracentrifugation as described above. Pellets were then washed in PBS and resuspended in PBS for DNA extraction. To remove free DNA and DNA associated with vesicles, the purified S13EV samples were treated with 30 μg/ml of both DNase and RNase A at 25°C overnight, followed by heat inactivation of the nucleases at 70°C for 10 min. Parental cell genomic DNA and nuclease‐treated S13EVs were extracted using an Exgene DNA extraction kit (Gene All, Korea) and a QIAamp MinElute Virus Spin Kit (Qiagen, USA), respectively, according to the manufacturer's instructions. The concentration of all genomic DNA was determined using a NanoDrop 2000 spectrometer (Thermo Scientific, Waltham, USA), and the genomic DNA was preserved at −20°C. PCR amplification of 16S rRNA, *pro*, *blh* (15,15′‐*β*‐carotene dioxygenase), and *crtI* (phytoene dehydrogenase) genes was conducted according to a previously described method (Kwon et al., 2016). The amplified products were separated by agarose gel electrophoresis.

### 2.8 Sampling, purification, sequencing, and analysis

Surface seawater samples (800 L) were collected from Pohang Bay (36°2′50.80′′N, 129°22′37.21′′E) of East Sea on October 08, 2013. Water samples were immediately passed through a 20‐μm‐pore size mesh prefilter, followed by sequential filtration through a series of membrane filters (Advantec, Japan) of decreasing porosities (3 μm to 0.2 μm) to remove bacteria, eukaryotes, and large particles. The water sample filtrate was concentrated and purified by the same method as that described above for vesicle isolation, and DNA was extracted using a QIAamp MinElute Virus Spin Kit after treatment with DNase and RNase A. Sequencing was performed using an Illumina MiSeq platform, and the average library size ranged from 200 to 800. Paired‐end raw reads with a quality score of <Q25 were excluded from analyses. Sequencing data of raw read lengths of 9 and 20 Mbp were generated for cellular (0.2–3 μm) and vesicular DNA samples, respectively. *De novo* assembly of high‐quality paired‐end reads was assembled by IDBA‐UD (Peng, Leung, Yiu, & Chin, 2011) using default parameters. Data analysis was performed by the bioinformatics service at ChunLab Inc., Seoul, Korea. The Whole Genome Shotgun projects have been uploaded to both the IMG/MER (Project ID: Ga0068515) and MG‐RAST (Project ID: 4,600,660.3) servers. To determine the taxonomic distribution of environmental sequences in the datasets, all reads were subjected to BLASTX searches against the NCBI nr database using an E‐value cutoff of >10^−5^, and the reads were then phylogenetically assigned according to LCA‐based algorithms implemented in MEGAN (Huson et al., 2007). The data were compared using a maximum E‐value of 1e^−5^, a minimum identity of 60%, and a minimum alignment length of 15 bp. Further analysis of the taxonomy table generated at each taxa level was analyzed in a MS‐office excel datasheet.

## 3 RESULTS AND DISCUSSION

### 3.1 EV production

**Figure 1:**
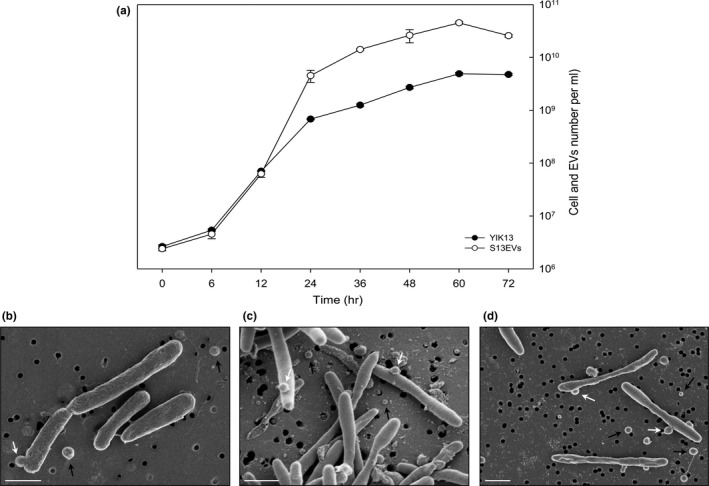
Parental cell growth (*Sediminicola* sp. YIK13) and vesicle production. (a) Number of parental cells (black circles) and S13EVs (open circles). SEM images of parental cells (*Sediminicola* sp. YIK13) after 6 hr (b), 24 hr (c), and 72 hr (d) of incubation. Some vesicles protruded from parental cells (white arrows), and others were released from the parental cell surface (black arrows). Scale bars: 1 μm

We investigated EV production using an isolated bacterial strain, *Sediminicola* sp. YIK13, which is known as a PR‐containing flavobacterium (Kwon et al., 2016). EVs produced by *Sediminicola* sp. YIK13 (S13EVs) appeared to be released continuously and spontaneously into the liquid medium under optimal growth conditions of the parental cells without the presence of any disturbing factors (e.g., antibiotics, UV irradiation, temperature, or nutrient stress) (Figure 1a). We observed S13EV production using the qNano method after 6 hr of culture growth, reaching a maximum of 4.5 × 10^10^ ml^−1^ when parental cell growth stabilized at 4.9 × 10^9^ ml^−1^. The ratio of the S13EV number to the total parental cell number increased approximately 10 fold between the late exponential phase (24 hr after incubation) and the stationary phase. This result is consistent with results reported for other bacteria (Biller et al., 2014; Mug‐Opstelten & Witholt, 1978). To morphologically confirm the EV production during cell growth, cells were collected at the lag, exponential, and stationary growth phases and were observed by scanning electron microscopy (SEM) (Figure 1b–d). These micrographic images revealed numerous lumen structures protruding from the surface of the parental cell (white arrows), which eventually migrated into the surrounding milieu as free EVs (black arrows). We did not observe any potential prophage or gene transfer agents in the complete genome sequence of this strain (Kwon & Kim, 2016).

### 3.2 EV size distribution and observation

**Figure 2:**
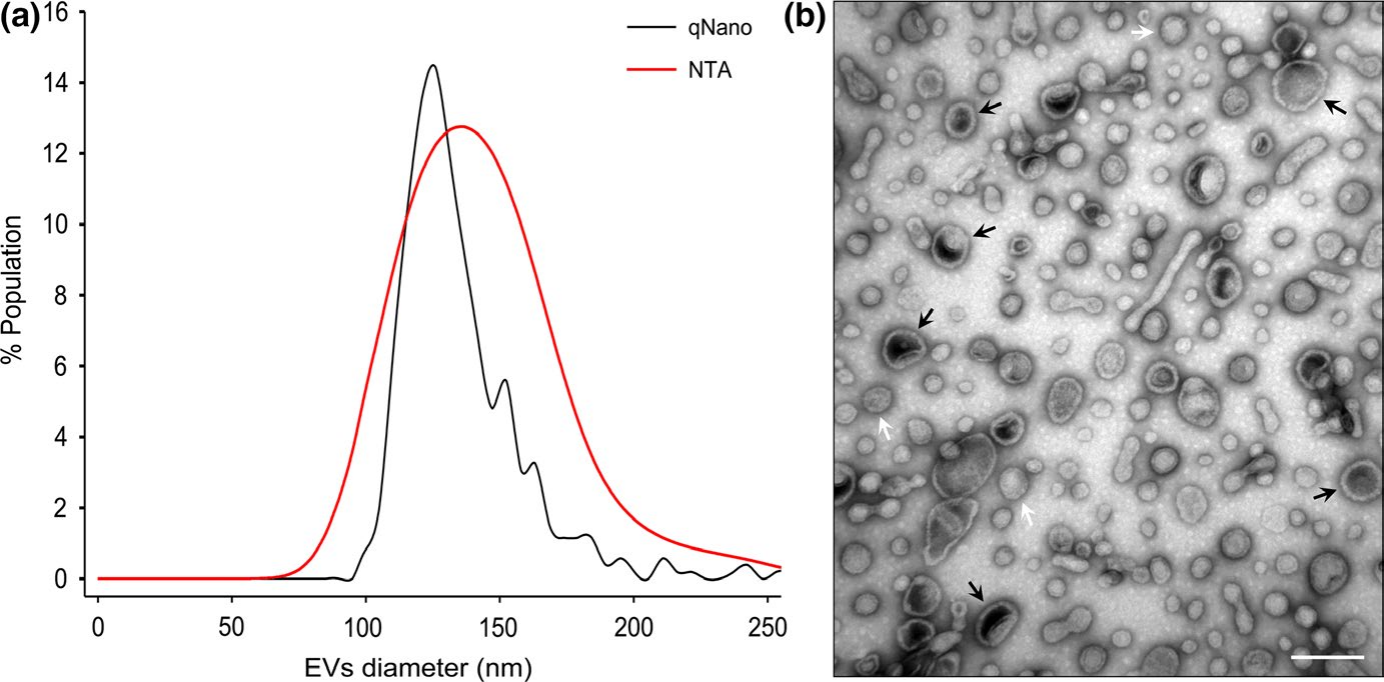
Size distribution of S13EVs and electron micrography image of S13EVs isolated from *Sediminicola*sp. YIK13 and stained with uranyl acetate. (a) S13EV size distribution estimated by qNano (black line) and NTA (red line) (see Materials and Methods). (b) Negatively stained S13EVs are visible in TEM images. See Choi et al. (2015) for a description of materials and methods used. Most vesicles have a spherical shape with a monolayer (white arrows) or bilayer membrane (black arrows) enclosing the electron‐dense substance within the core region. Parental cells were not observed. Scale bar: 100 nm

**Figure A1:**
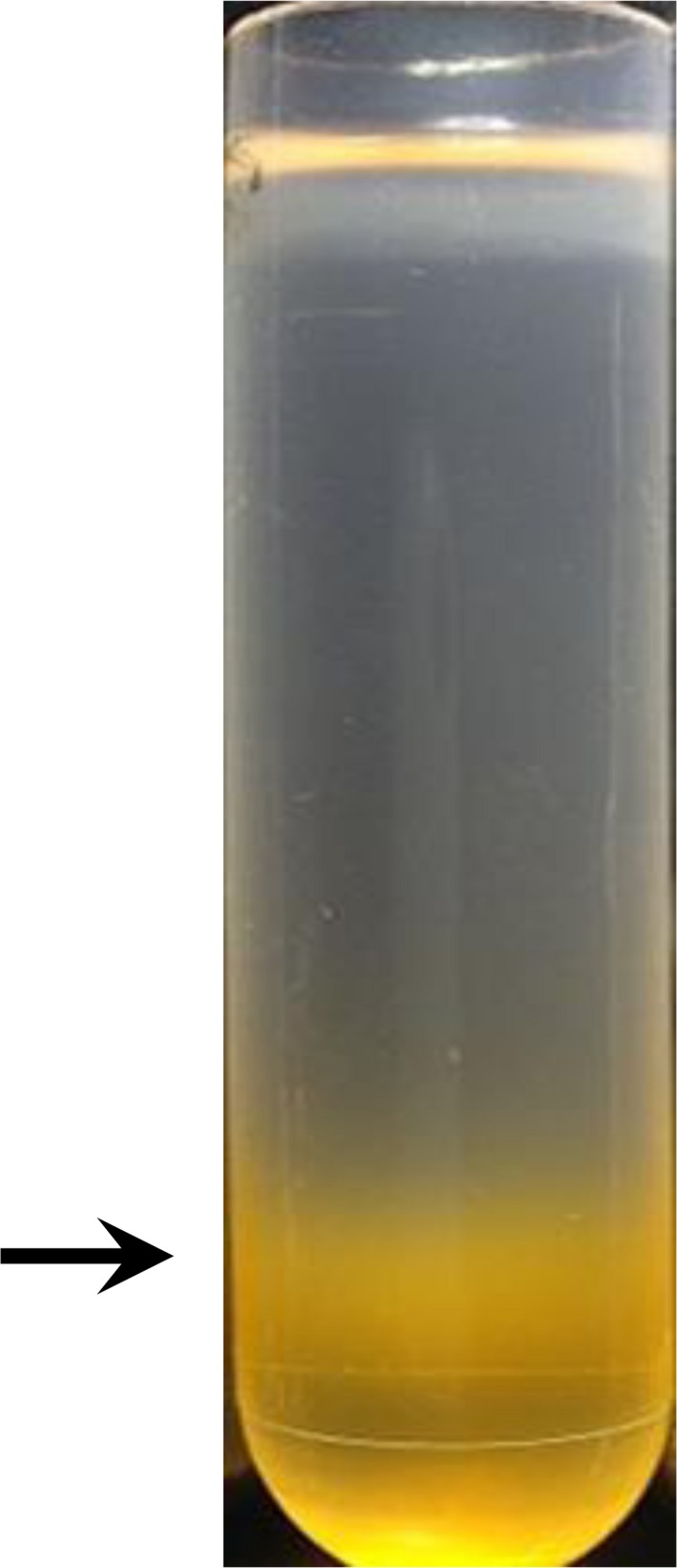
CsCl density gradient ultracentrifugation of the S13EVs band (arrow). The band shows the color of carotenoid

We concentrated and purified vesicles from 5 L of YIK13 bacterial culture broth using previously described procedures (Choi et al., 2015). After the equilibrium CsCl density gradient ultracentrifugation, we obtained one visible band containing orange pigment identical to the typical parental cell color, with a buoyant density ranging from 1.3282 to 1.3505 g/cm^3^ (Figure A1). Vesicles in this band were collected, and the level of parental cell contamination in the purified S13EV samples was verified by parental cell colony recovery using nutrient agar as well as direct transmission electron microscopy (TEM) observation. The size and number of purified S13EVs were measured by two methods: qNano and nanoparticle tracking analysis (NTA) (see Materials and Methods). The sizes were determined to be ranging from 88 to 250 nm and 50 to 250 nm using the qNano and NTA methods, respectively (Figure 2a). qNano vesicle counts were approximately 5% lower than NTA vesicle counts for vesicles >90 nm in diameter; moreover, the average S13EV diameter was 130 nm. A combination of methods to more accurately characterize EVs is likely required to minimize the differences in quantification (Erdbrügger & Lannigan, 2016). After negative staining, we examined S13EV structures via TEM. Most S13EVs had a spherical structure consisting of a monolayered membrane (white arrows, approximately 89.1%, *n = *841) (Figure 2b). However, some vesicles had a bilayered membrane (black arrows, approximately 10.9%) with an electron‐dense substance, like the bilayer structure (containing cytoplasmic material), produced by several gram‐negative bacteria—called outer‐inner membrane vesicles, as reported by Pérez‐Cruz et al. (2013), Pérez‐Cruz et al. (2015). Recently, Pérez‐Cruz et al. (2015) discovered that MVs, designed as outer‐inner membrane vesicles, represent only 1.2%, 0.54%, and 0.23% of the total number of vesicles produced by three pathogenic bacteria, *Neisseria gonorrhoeae*, *Pseudomonas aeruginosa* PAO1, and *Acinetobacter baumannii* AB41, respectively.

### 3.3 Characterization of PR‐containing EVs

**Figure 3:**
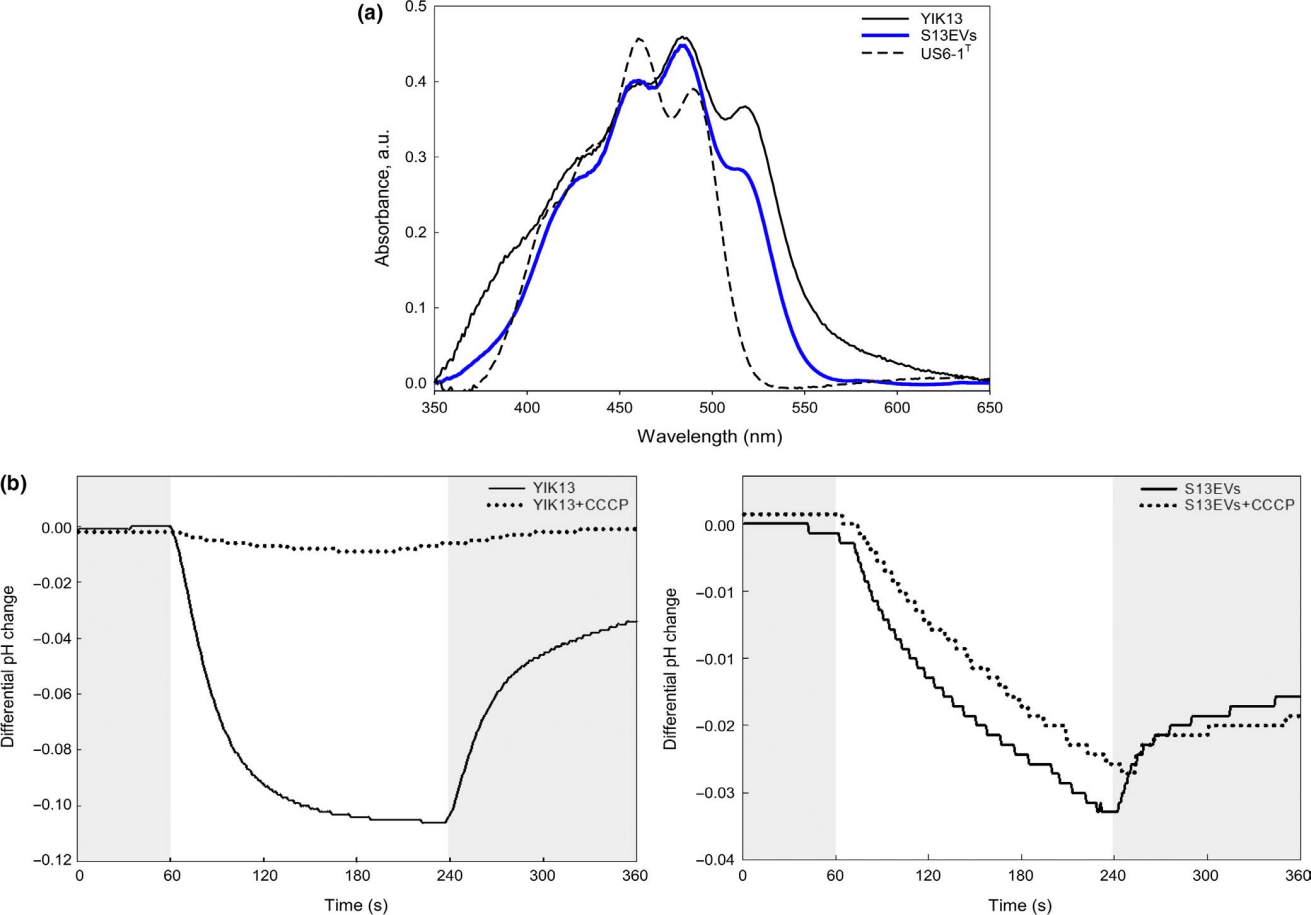
Absorption spectra (a) and light‐induced pump activities of native YIK13 cells and purified S13EVs (b). (a) Absorption spectra showing carotenoids (423, 457, and 484 nm) and PR (518 nm) from parental cells (black line) and S13EVs (blue line). *Novosphingobium pentaromativorans* US6–1^T^, which does not contain PR but contains carotenoids, was used as a negative control (dashed line). (b) Pump activity of parental cells (left panel) and S13EVs (right panel). CCCP was added to samples as a control. Solid and dotted lines: without and with 10 μmol/ml CCCP, respectively. Changes in pH (initial pH: 7.0–7.2) were monitored in dark (gray regions) and light (>440 nm, white region) conditions in triplicate measurements

**Figure 4:**
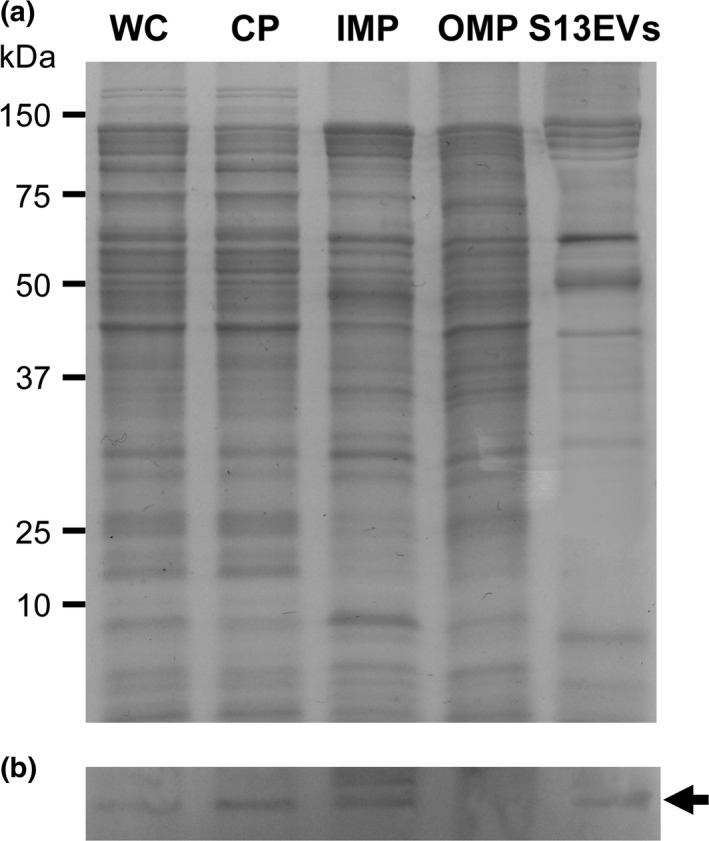
Coomassie Brilliant Blue‐stained SDS‐PAGE (a) and Western blot analyses (b) for the presence of PR in whole‐cell lysate (WC), cytoplasmic proteins (CP), inner membrane proteins (IMP), outer membrane proteins (OMP), and S13EVs. The blot was probed with an anti‐PR monospecific polyclonal antibody (targeting peptide PR_Arg71‐Tyr86_). Arrow indicates the PR protein band

To investigate whether 13EVs possess PR, we measured the absorption spectra of the purified S13EV. Results indicated that both putative PR and carotenoids, such as *β*‐carotene, absorbed at 518 nm and were likely dominant pigments in both parental cell membranes and S13EVs (Figure 3a). The PR‐derived peak, however, was not detected in the negative control (i.e., *Novosphingobium pentaromativorans* US6‐1^T^), which contains carotenoids but not PR. Functional PR requires retinal covalent binding, which is synthesized from *β*‐carotene (Teramoto, Takaichi, Inomata, Ikenaga, & Misawa, 2003). We found the gene *crtEBIY* predicted to encode enzymes needed to synthesize *β*‐carotene from farnesyl diphosphate and isopentenyl diphosphate in the parental cell genome sequences (Kwon & Kim, 2016). Furthermore, to understand whether and how S13EV PR proteins function, we measured the activity of the S13EV PR light‐driven proton pump and found that S13EVs have light‐driven pumping activity like that of the parental cells (solid lines in Figure 3b). However, S13EV pumping activity (right panel in Figure 3b) was not completely inhibited when S13EVs were treated with a protonophore CCCP, whereas parental cell production was completely inhibited (left panel in Figure 3b). We surmised that only bilayered EVs are effective for the proton‐pumping function and the PR embedded in the monolayered membrane cannot form the electrical signals to generate a membrane potential. Although this uncoupling agent effect was an unexpected result that has not yet been completely clarified, we observed that light‐induced activity associated with S13EVs has the PR proton‐pumping function. To further examine whether PR proteins of parental cells are packaged within these S13EVs, parental cell and EV extracts were subjected to Western blot analyses using an anti‐PR peptide‐specific antibody. The SDS‐PAGE of proteins extracted from the whole cells, cytoplasmic, inner membrane, outer membrane of the parental cell, and EVs (Figure 4a) indicated that EV protein profiles show different and simpler patterns compared with other subcellular localizations. These results are consistent with those of previous studies (Bauman & Kuehn, 2006; Choi et al., 2011; Renelli et al., 2004; Tashiro et al., 2010). We detected PR protein from all the extracts except for the outer membrane protein of parental cells at expected sizes, that is, approximately 27 kDa (Figure 4b). These results demonstrated that PR associated with EVs originated from the parental cells.

Light‐activating, PR‐induced proton‐motive force drives ATP synthesis as protons move into or out of the cell through an ATP synthase complex (Béjà et al., 2001, 2000; Martinez et al., 2007). We measured light‐induced ATP production in the parental cells and S13EVs, which exhibit proton ion‐pumping activities, using a luciferase‐based assay method. The ATP synthesis of the parental cells (final concentration of 2 × 10^7^ cells per well, grown to the late stationary phase) was increased to 37.1% ± 5.8% (mean ± standard deviation) after light exposure compared with dark conditions but did not detect the light‐dependent ATP production in the S13EVs (final concentration of 2 × 10^9^ per well) or the presence of ATP in the S13EVs without light exposure (data not shown). To understand the actual light‐induced pump activity of S13EVs under the experimental condition, a quantitative estimation of the proton transport activity by measuring the PR absorbance, ∆pH, and ATP production was attempted for S13EVs based on the study by Yoshizawa, Kawanabe, Ito, Kandori, and Kogure (2012). Although ∆pH for the specimens was measureable as shown in Figure 3, the amount of ATP produced by the PR‐mediated proton‐motive force in S13EVs was estimated to be insufficient for the ATP determination ranging between 10^−20^ and 10^−13^ moles (Table A1).

**Figure 5:**
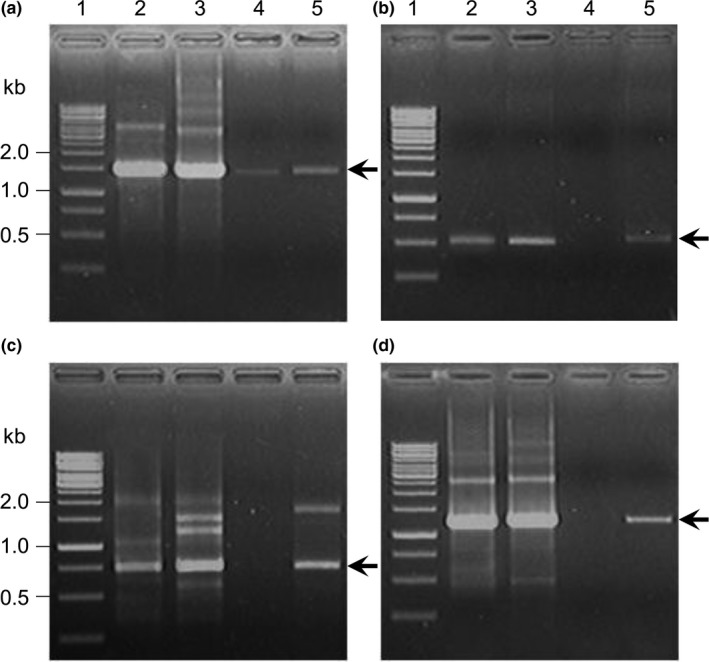
Electrophoretic analysis of PCR products of 16S rRNA (a), *Pro* (b), *Blh* (c), and *CrtI* (d) genes amplified from the genomic DNA of parental cells and EVs. Lane 1, 1‐kb DNA ladder; lane 2, AKS622^T^ gDNA; lane 3, YIK13 gDNA; lane 4, AKS622^T^‐derived EV DNA; lane 5, S13EV DNA. Arrows indicate relevant amplicon products, and molecular sizes are on the left

**Figure A2:**
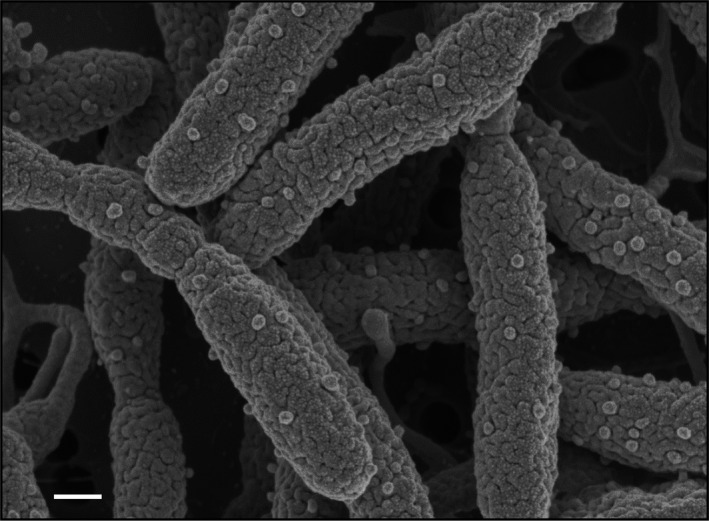
SEM image of vesicles protruding from the cell surface of Nonlabens antarcticus AKS622T. Scale bar: 200 nm

To examine the presence of genes required for retinal biosynthesis within vesicles, we collected DNA from nuclease‐treated S13EVs. We amplified the genes encoding 16S rRNA and *pro*, including genes such as *blh* and *crtI* involved in retinal production (Misawa et al., 1995; Sabehi et al., 2005). The PCR products showed that S13EVs have gene sequences identical to those in the parental cell (Figure 5). To compare whether these genes are packaged within vesicles derived from bacteria inhabiting in different environments, we selected the PR‐containing marine psychrophilic flavobacterium, *Nonlabens antarcticus* AKS622^T^, in addition to *Sediminicola* sp. YIK13 (Kwon et al., 2016, 2014). SEM analyses clearly showed the presence of numerous small EV‐like structures (40–90 nm) protruding from the surface of the AKS622^T^ cells (Figure A2). We attempted to amplify the same genes from the AKS622 vesicle‐DNA. The products, however, only contained the 16S rRNA gene sequence (Figure 5). This result suggests that the type of PR‐related parental genes packaged into EV depend on the types of bacteria species and culture conditions (Kadurugamuwa & Beveridge, 1995; Schwechheimer & Kuehn, 2015).

Results discussed above imply that some, but not all, PR‐containing bacteria can produce vesicles containing PR and carotenoid genes in the vesicular membrane. For example, *Sediminicola* strain YIK13 is capable of this, but not *N. antarcticus* AKS622^T^. S13EVs have mechanisms that form the proton‐motive force gradient for light‐driven proton pump activity; however, the amount of ATP produced was too small to be substantially determined. Although the PR‐containing bacterium *Sediminicola* sp. YIK13 can produce vesicles containing PR‐related genes, whether the entire PR‐containing bacteria produce EVs containing PR‐related genes in natural environments remains uncertain. EVs that contain PR‐related genes and are produced and distributed in natural environments may play an important role in PR gene horizontal transfer in the natural environments.

### 3.4 Distribution of microbial rhodopsin gene‐containing EVs in natural environments

Using a metagenomic approach, we investigated the distribution of microbial rhodopsin‐containing EVs from a coastal environment in Pohang Bay. Surprisingly, EV samples purified from Pohang Bay surface water contained diverse DTE (highly conserved residues for H^+^ pumps in helix C) motif‐containing rhodopsin genes, thus indicating the presence of PR, xanthorhodopsin, and actinorhodopsin (Table 1 and A2). Retinal biosynthetic pathway genes were also found in the metagenomics data of these EV samples. The closest taxonomic groups of the microbial rhodopsin, *β*‐carotene, and retinal synthesis genes in the DNA sequences of EVs collected from the bay were Proteobacteria, Bacteroidetes, Firmicutes, and Actinobacteria, whereas the most dominant was the alphaproteobacteria SAR11 group. The dominant bacterial phyla, Proteobacteria (61%), Bacteroidetes (21%), Actinobacteria (12%), and Firmicutes (2.6%) in Pohang Bay are consistent with those for major EVs containing rhodopsin genes or retinal biosynthetic pathway genes (Table 2). This result supports the observation that microbial rhodopsin‐containing organisms are likely to produce EVs in natural environments. PR‐containing bacteria constitute 13%–70% of all microorganisms (light‐harvesting bacteria) in oceanic surface waters (Martinez et al., 2007; Venter et al., 2004), which are present in three major marine classes: Alphaproteobacteria, Gammaproteobacteria, and Flavobacteria. Several studies have demonstrated that PR and chromophore genes have been acquired through horizontal gene transfer between related and unrelated lineages (Gómez‐Consarnau et al., 2010; McCarren & DeLong, 2007). However, no study has demonstrated the presence or involvement of an agent that transfer these genes. Several research groups have determined that OMVs and MVs, similar to EVs, carry DNA and transfer it via vesicles to other cells through what is assumed to be a fusion reaction (Gaudin et al., 2013; Renelli et al., 2004; Yaron et al., 2000).

**Table 1:** Distribution of microbial rhodopsin, *β*‐carotene, and retinal synthesis genes in the DNA sequences of EVs collected from Pohang bay

Accession (gi)	Protein description	COG or GO ID	Length (amino acid)	Coverage	Closest taxonomy	
					Phylum	Subcategory
Microbial rhodopsin type						
56,144	Proteorhodopsin	COG5524	256	19	*Proteobacteria*	*SAR11*
157,357			103	5	*Bacteroidetes*	*Flavobacteriales*
172,205			121	4	*Bacteroidetes*	*Flavobacteriales*
183,919			115	5	*Proteobacteria*	*SAR11*
131,148	Xanthorhodopsin	COG5524	148	6	*Proteobacteria*	*SAR86*
132,188			148	6	*Proteobacteria*	*Methylophilales*
156,148			130	5	*Proteobacteria*	*Methylophilales*
171,194	Actinorhodopsin	COG5524	121	7	*Actinobacteria*	*Planktophila*
Synthesis of *β*‐carotene						
121,938	Farnesyl‐diphosphate synthase (*ispA*)	COG0142	133	4	*Proteobacteria*	*SAR11*
148,296			135	5	*Proteobacteria*	*SAR11*
133,712	Phytoene synthase (*crtB*)	COG1562	147	10	*Proteobacteria*	*SAR11*
48,707	Phytoene desaturase (*crtI*)	COG1233 or GO:0,009,055; GO:0,015,979; GO:0,015,995; GO:0,016,117; GO:0,016,491; GO:0,055,114	199	12	*Planctomycetes*	*Planctomycetales*
108,888			172	9	*Proteobacteria*	*SAR11*
128,923			151	6	*Proteobacteria*	*SAR11*
153,543			52	8	*Proteobacteria*	*SAR11*
156,985			85	6	*Bacteroidetes*	*Flavobacteriales*
182,462			69	8	*Proteobacteria*	*SAR11*
201,719	Lycopene *β*‐cyclase (*crtY*)	COG0654	107	5	*Bacteroidetes*	*Flavobacteriales*
191,630	15,15′‐*β*‐Carotene dioxigenase (*blh*)	GO:0,016,021	112	8	*Proteobacteria*	*SAR11*

**Table 2:** Taxonomic abundance of sequences from vesicular (<0.2 μm) and bacterial (0.2–3 μm) fractions in the surface water of Pohang Bay

Superkingdom	Phylum	Sequence count	
		Vesicles (<0.2 μm)	Bacteria (0.2–3 μm)
Bacteria	*Proteobacteria*	31,406	67,067
	*Bacteroidetes*	5,128	23,236
	*Firmicutes*	4,406	2,871
	*Actinobacteria*	1,337	12,956
	*Planctomycetes*	1,049	697
	*Cyanobacteria*	830	1,049
	*Aquificae*	415	188
	*Synergistetes*	360	104
	*Spirochaetes*	289	367
	*Deferribacteres*	271	98
	*Deinococcus‐Thermus*	172	162
	*Acidobacteria*	144	–
	*Chloroflexi*	136	439
	*Verrucomicrobia*	122	177
	*Tenericutes*	105	59
	*Chlorobi*	103	261
	*OD1*	79	50
	*Fusobacteria*	47	76
	*Cloacamonas*	44	20
	*Nitrospirae*	44	86
	*Thermotogae*	22	175
	*Lentisphaerae*	21	32
	*TM7*	15	13
	*Chrysiogenetes*	12	12
	*Thermodesulfobacteria*	10	14
	*Elusimicrobia*	8	16
	*Poribacteria*	6	28
	*Armatimonadetes*	4	–
	*Fibrobacteres*	3	5
	*Gemmatimonadetes*	3	20
	*Dictyoglomi*	2	21
	*WS3*	2	3
	*OD9*	—	6
	*OD11*	—	3

Based on the results of the present study, we concluded that PR‐containing bacteria can release EVs containing both light‐driven proton pump activity and PR‐related genes into the surrounding environment. EVs in natural environments may be a potential agent that biologically transfer PR and chromophore retinal genes and thus play an important role as a driving force for horizontal gene transfer in natural environments.

## 4 CONCLUSION

This study is the first to demonstrate the production of two types of spherical vesicles (S13EVs) that were composed of mono‐ and/or bi‐layered membranes residing the *pro* and retinal synthesis genes in the culture of PR‐containing *Sediminicola* sp. YIK13. The S13EVs demonstrated light‐induced pump activity identical to that demonstrated by the parental cells. PR‐containing *Sediminicola* sp. YIK13 could produce EVs containing the *pro* gene and retinal synthesis genes, whereas the PR‐containing marine flavobacterium *N. antarcticus* AKS622^T^ was incapable of producing EVs. Metagenomic analyses suggest that EVs serve as both a reservoir and an agent for transferring diverse microbial rhodopsin genes toward the microbial community in the marine systems. It remains to be determined whether S13EVs indeed function as an agent for horizontal gene transfer. To answer this question, an investigation is in progress, which might elaborate on MVs' function accomplishing the gene flux in the environment.

## CONFLICT OF INTEREST

The authors declare no conflict of interest.

## AUTHORS CONTRIBUTION

YMK performed all experiments, data analysis, and manuscript finalization. AKP performed additional metagenomics analysis and interpretation. HXC participated in the design and discussion of this study. SJK oversaw the project and was responsible for finalizing the manuscript. All authors have read and approved the manuscript.

## ETHICS STATEMENT

None required.

## Data Availability

All data are included in the article.

## APPENDIX 1

### 1.1

[Correction added on 14 March 2019, after first online publication: the figures and tables that were in Supporting Information have been included in the Appendix section of the article].

**Table:** Table A1 The quantification of PR molecular and ATP produced by light‐induced proton pumping activity in S13EVs

	pH		Δ[H+]		Involved PR molecule	Predicted membrane area μm^2^	Effective (#) & proportion of the employed sample			Predicted ΔATP amount		
	Initial	Final	Mole	Ion			#	%	100%	Molecule	Mole	nmole
S13EVs	7.2	7.18	3.61 × 10^−9^	2.17 × 10^15^	1.75 × 10^13^	6.74 × 10^8^	7.93 × 10^10^	39.7%	2 × 10^11^	5.43 × 10^14^	9.03 × 10^−10^	0.90

Calculation is based on Fig. 3B, whose ΔpH determination range was 7.0–7.2 and supposing initial pH was 7.20.

The proportion of S13EVs was based on measured concentration of 2 × 10^11^ as 100% and average surface area of S13EVs is 0.289 μm^2^.

Detailed procedures for these calculations were based on a previous description (Yoshizawa et al., 2012).

**Table:** Table A2 Microbial rhodopsin sequences identified in EV fractions

		Nucleotide sequence	Identity	Best hit; GeneBank
PR	gene_id_56144	ATGAAAAAACTTAAATTGTTTGCGCTTACAGTTGTAGCCCTAATGGGTGTTACAGGTGTTGCAAACGCAGAGACAGTGATGTTAGCTCAGGATGATTTCGTTGGTATTTCATTTTGGTTAGTATCAATGGCTTGTTTAGCTGCTACTGTGTTCTTTTTCTTAGAAAGAAGTTCAGTTCCAGCTGGTTGGAGAGTTTCAATGACAGTTGCTGGTCTAGTAACTGGTATTGCATTCGTACACTACATGTACATGAGAGATGTATGGATCATGACTGGTGACTCACCAACTGTATACAGATACATTGACTGGTTAATTACAGTGCCATTACTAATGTTAGAATTCTATTTTGTTCTATCAGCAGTAAACAAAGCAGACTCAGGAATTTTCTGGAGATTAATGCTTGGTACATTAGTAATGCTAGTAGGTGGATACTTAGGAGAAGCAGGATACATCAACGCTACACTTGGTTTCATTATCGGTATGGCTGGTTGGGTATACATTCTTTATGAAGTATTCTCAGGTGAAGCAGGTAAGAGAGCAGCGAAAAGTGGTAATAAAGCACTTGTAACTGCTTTTGGTGCAATGAGAATGATCGTTACTGTAGGTTGGGCTATTTACCCATTAGGTTACATTTTTGGTTACCTAACAGGTGGTGTAGATGCTAACTCACTAAACGTGATTTACAACGCAGCTGACTTCTTGAACAAGATCGCTTTCGGTCTGATCATTTGGGCAGCAGCAATGCAACAACCTGGTAGAGCTAAGTAG	93%	Alpha proteobacterium HIMB5; CP003809
	gene_id_157357	AACGGTCCTGCATTATGGGGTCTTATCTCTGGTTTGGCTTATTTCGCTATCGTTTATGAAATCTGGAAAGGTGGAGCTGCCAAGTTGGCTGAAGCTGCTGGTGGAGCCGTTCTTTCTGCTCACAAAACACTTTTGAAGTTTGTATTCATAGGATGGGCGATCTATCCTATCGGATACATGGCCGGTACTGAAGGATGGTACAGCGGAATCTTTGGTGGTCTTCCCATGGACGTTATCTACAACGTTGGTGATGCCATCAACAAAATTGGATTCGGTTTGGTTGTTTACAACCAGCTGTTAGTTCTAAGTAATCTTAAAGACAGCAGTTCAACTGCTCAAGATAAAAGAATCGCTCTTCGGAGCGATTTTTTTTTACATGCTATCGGG	100%	Uncultured microorganism clone 0fde9d2a54827e3155af81687fa2971e; KJ928528
	gene_id_172205	TATGTAGGGTTCACTTTTTTTGTAGGTTGTATGGCAATGATGGCAGCATCAGCGTTTTTCTTCCTGTCTATGAACTCTTTCGACAACAAATGGAGAACCTCCATTTTGGTATCAGGTTTGATTACCTTTATCGCTGCAGTACACTACTGGTACATGAGAGACTATTGGGCAACTAACGCCACCTCACCAACATTCTTCCGTTATGTAGACTGGGTGTTGACCGTACCATTAATGTGTGTAGAGTTTTACCTTATTCTAAAGGCGGCTGGAGCCACTAAATCATTGATGTGGAAACTAATTTTCCTATCTGTAATCATGTTAGTAACAGGTTACTTTGGAGAAGCAGTTCACACTACCGGTAAC	92%	Uncultured bacterium clone W1‐7; EU683552
	gene_id_183919	GTTGCCCTAATGGGTGTTACAGGTGTAGCAAACGCAGAAACTGCCATGTTAGCTCAAGATGATTTCGTAGGTATTTCATTTTGGTTAGTATCAATGGCTTGTTTAGCTGCTACTGTATTCTTTTTCTTAGAAAGAAGTTCGGTTCCAGCTGGATGGAGAGTTTCAATGACTGTTGCTGGTCTAGTAACTGGTATTGCATTCGTACACTACATGTATATGAGAGATGTATGGATCATGACTGGTGACTCACCAACTGTCTACAGATACATTGACTGGTTAATCACAGTGCCATTACTAATGTTAGAATTCTATTTTGTTCTATCAGCAGTAAACAAAGCAGATTCA	97%	Bacterium HTCC8038; EF616637
XR	gene_id_131148	ACTCTTTTAGCAGCTCTCTCTGCTGACGGGAGAGATCAGGTTTATTTGAAACAAGCTCTTGTTTCTGAAACCGCTGTTAACATCATTGCAAGTGTGACTTACTATTACTTTATGATCTATCTTTATGCCGATAAATTAACATTGAAGAACGTAACACCTATAAGATATCTTGATTGGGCTTTCACAACACCCCTACTTCTACTGTCTTTTGTACTTATTTCTTCCTACGGATCCAACAAAGGTCAATCTGTTGATTTAGAATATTTGATATATATCATTATACTCAATCTTGGTATGTTGTACTTCGGTTATATAGGAGAAAAGGGAATAATGAATTTCTGGATTGCATTTATCCTAGGATTTGCTTGTTATGCAGGTCTCATATATCTACTCTGGGACCAGTACATAGATAAAGAAGATGAGGGTTCAAAAACCCTGTTCTATAT		35% identity only on the protein level to predicted rhodopsin EU915778 from uncultured microorganism clone PF97522
	gene_id_132188	GTCTTAGTGATGAGACTCTCAAAAGAGGAAACATATTCTAAAGGTACAAAATTAGCTTTAGCTGCTGCAGTTATGGTCATCTTAGGTTACCCAGGTGAAGTAATTACTGATCCATCAATGTACGGTGATCGTTGGATGTGGTGGGTACTAGCTATGATTCCATTTGTATACATCGTTTGGGATCTAGTTAGCGGCCTTGGCGCATCAATCAGCAAACAGCCAAAAGCAGTTCAAGGTCTTGTAAGTAAAGCAAGAACTTTAACAATTTTGTCATGGTGCTTTTATCCAATCGTATTCGTTTTCCCAATGATTGGTTTACAAGCAGACCCAGGTGCTGTTAATCCTGCAATTCAAGTAGGTTACACAGTTGCTGACGTGGTTGCAAAAGCACTATTTGGTGTAATGATTTATATGATTGCTCAGCGTAAGTCTGATGCAGGTGCAT	96%	Methylophilales bacterium MBRSG12; CP011003
	gene_id_156148	AAATGGAGAATCTCTATGGAAGTAGGCGGATATAATTTAGTCTATAACTCACTGTCATTCGCAATTGCAACATTTGGTGCGGCTACAGTATTCTTTTTTGCTCAGCGTTCTCAAGTCGCTCCAGCATACAAAACTGCGCTATCCCTATCAGGGCTAGTTTGTTTAATCGCTTTTTATCATTATCTAAGAATGTTTGAAAGTTTCAATGATGCTTACACATTAGTTAATGGTACTGTTGAAGCAACAGGTGCTCAATTCAATGACGCATATCGTTACGTGGATTGGTTACTAACTGTGCCTCTTTTACTCCTTGAGTTAGTCTTAGTGATGAGACTGTCAAAAGAGGAAACATATTCTAAAGGTACAAAATTAGCTTTAGCTGCTGCAGTT	97%	Methylophilales bacterium MBRSG12; CP011003
ActR	gene_id_171194	TTTGGACCAGATGCTGTTGCTGGAGCCTCCTACAACGTTGGATACCGCTACGTTGACTGGTTCCTAACTGTTCCACTGCTACTGGTTGAGACCGTCGCAGTTCTCGCGCTAGCTCGCCAGGCACAGAGCTCACTGCTCGTCCGCCTAGTACCAGCAGCAGCCCTGATGATCGCACTGGGTTACCCAGGAGACTCCGGCATGACCATGGGCCTAGCACCATCGGTTTGGGGTCTACTATCGACCATCCCATTCCTCTACATCCTCTACGTACTGTTCGTTGAGCTCGGCAAGAGCCTCGAGCGTCAGAGCGAGGCTGTACAGCGCAAGATCAAGGAGCTACGTCTTCTTCTTCTCGCGACCTGG	97%	Uncultured bacterium clone OTU18; HF571309
